# Feasibility and effectiveness of transcutaneous auricular vagus nerve stimulation (taVNS) in awake mice

**DOI:** 10.1111/cns.70043

**Published:** 2024-09-11

**Authors:** Yu‐Mei Yu, Rui Yao, Zhou‐Liang Liu, Yao Lu, Yang‐Zi Zhu, Jun‐Li Cao

**Affiliations:** ^1^ Jiangsu Province Key Laboratory of Anesthesiology Xuzhou Medical University Xuzhou Jiangsu China; ^2^ Jiangsu Province Key Laboratory of Anesthesia and Analgesia Application Technology Xuzhou Medical University Xuzhou Jiangsu China; ^3^ NMPA Key Laboratory for Research and Evaluation of Narcotic and Psychotropic Drugs Xuzhou Medical University Xuzhou Jiangsu China; ^4^ Department of Anesthesiology Xuzhou First People's Hospital Xuzhou Jiangsu China; ^5^ Department of Anesthesiology Xuzhou Central Hospital Xuzhou Jiangsu China; ^6^ Department of Anesthesiology Affiliated Hospital of Xuzhou Medical University Xuzhou Jiangsu China

**Keywords:** awake, effectiveness, feasibility, memory, transcutaneous auricular vagus nerve stimulation

## Abstract

**Aims:**

Transcutaneous auricular vagus nerve stimulation (taVNS) is widely used to treat a variety of disorders because it is noninvasive, safe, and well tolerated by awake patients. However, long‐term and repetitive taVNS is difficult to achieve in awake mice. Therefore, developing a new taVNS method that fully mimics the method used in clinical settings and is well‐tolerated by awake mice is greatly important for generalizing research findings related to the effects of taVNS. The study aimed to develop a new taVNS device for use in awake mice and to test its reliability and effectiveness.

**Methods:**

We demonstrated the reliability of this taVNS device through retrograde neurotropic pseudorabies virus (PRV) tracing and evaluated its effectiveness through morphological analysis. After 3 weeks of taVNS application, the open field test (OFT) and elevated plus maze (EPM) were used to evaluate anxiety‐like behaviors, and the Y‐maze test and novel object recognition test (NORT) were used to evaluate recognition memory behaviors, respectively.

**Results:**

We found that repetitive taVNS was well tolerated by awake mice, had no effect on anxiety‐like behaviors, and significantly improved memory.

**Conclusion:**

Our findings suggest that this new taVNS device for repetitive stimulation of awake mice is safe, tolerable, and effective.

## Graphical abstract

We developed a new taVNS method for safe, tolerable, and effective repetitive stimulation of awake mice. The administration of taVNS to awake mice for three consecutive weeks significantly improved memory but had no effect on anxiety‐like behaviors.

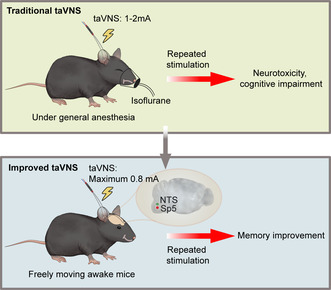

## 1 INTRODUCTION

Transcutaneous auricular vagus nerve stimulation (taVNS) is a noninvasive and nonpharmacologic treatment involving cranial nerve stimulation that has significant therapeutic effects and good clinical application value for various neurological and psychiatric disorders.^1^, ^2^, ^3^ The concept of taVNS was proposed in 2000, and more than 20 years of research has revealed that this strategy can treat over 30 diseases and disorders.^4^, ^5^ Specifically, taVNS, which takes advantage of the intrinsic plasticity of the nervous system, has been found to effectively treat refractory epilepsy,^6^, ^7^, ^8^, ^9^, ^10^, ^11^ major depression,^2^, ^12^, ^13^, ^14^, ^15^ migraine,^16^, ^17^, ^18^, ^19^, ^20^ cognition,^21^, ^22^, ^23^ and memory impairment.^3^, ^24^, ^25^, ^26^ taVNS has promising application value for clinical disease prevention due to its safety, tolerability, and effectiveness.

Research on the mechanism of action of taVNS has gradually progressed to include animal experiments, but there is still a significant gap between the stimulation methods used in animal research and clinical settings. Two of the important differences are the use of anesthesia and the stimulation intensity. In many clinical studies, taVNS is repeatedly administered for a long period to awake patients.^13^, ^18^, ^27^ In mice, repeated or prolonged general anesthesia is unavoidable due to the difficulty of attaching electrodes and performing nerve stimulation on awake mice,^28^ which can lead to neurotoxicity and cognitive impairment.^29^, ^30^, ^31^, ^32^ When an appropriate stimulation intensity is used, taVNS is well tolerated by patients even when repeated over a long period. Unfortunately, the high stimulation intensity tolerated by mice under general anesthesia was intolerable to conscious mice. Obviously, the design of a new stimulation method that completely mimics the technique used in clinical settings and can be well tolerated by awake mice is extremely necessary for generalizing research findings related to the effects of taVNS. The development of this method in animal studies avoids the interference of repeated general anesthesia to the experiment itself, which enables closer integration of basic and clinical research.

In this study, we utilized a taVNS device that could be reliably immobilized in mice to investigate the effects of repeated stimulation at an appropriate stimulation intensity in awake mice.

## 2 MATERIALS AND METHODS

### 2.1 Animals

Male C57BL/6J mice (at least 8 weeks old) were obtained from the Laboratory Animal Center at Xuzhou Medical University. The mice were housed in single cages at a stable temperature (22 ± 1 °C) and humidity (30%–50%) on a 12‐h light/dark cycle (starting at 7:00 a.m.) with ad libitum access to food and water. The care and use of animals and the experimental protocols used in this study were approved by the Institutional Animal Care and Use Committee and the Office of Laboratory Animal Resources of Xuzhou Medical University under the Regulations for the Administration of Affairs Concerning Experimental Animals (1988) in China.

### 2.2 Electrode implantation

**FIGURE 1:**
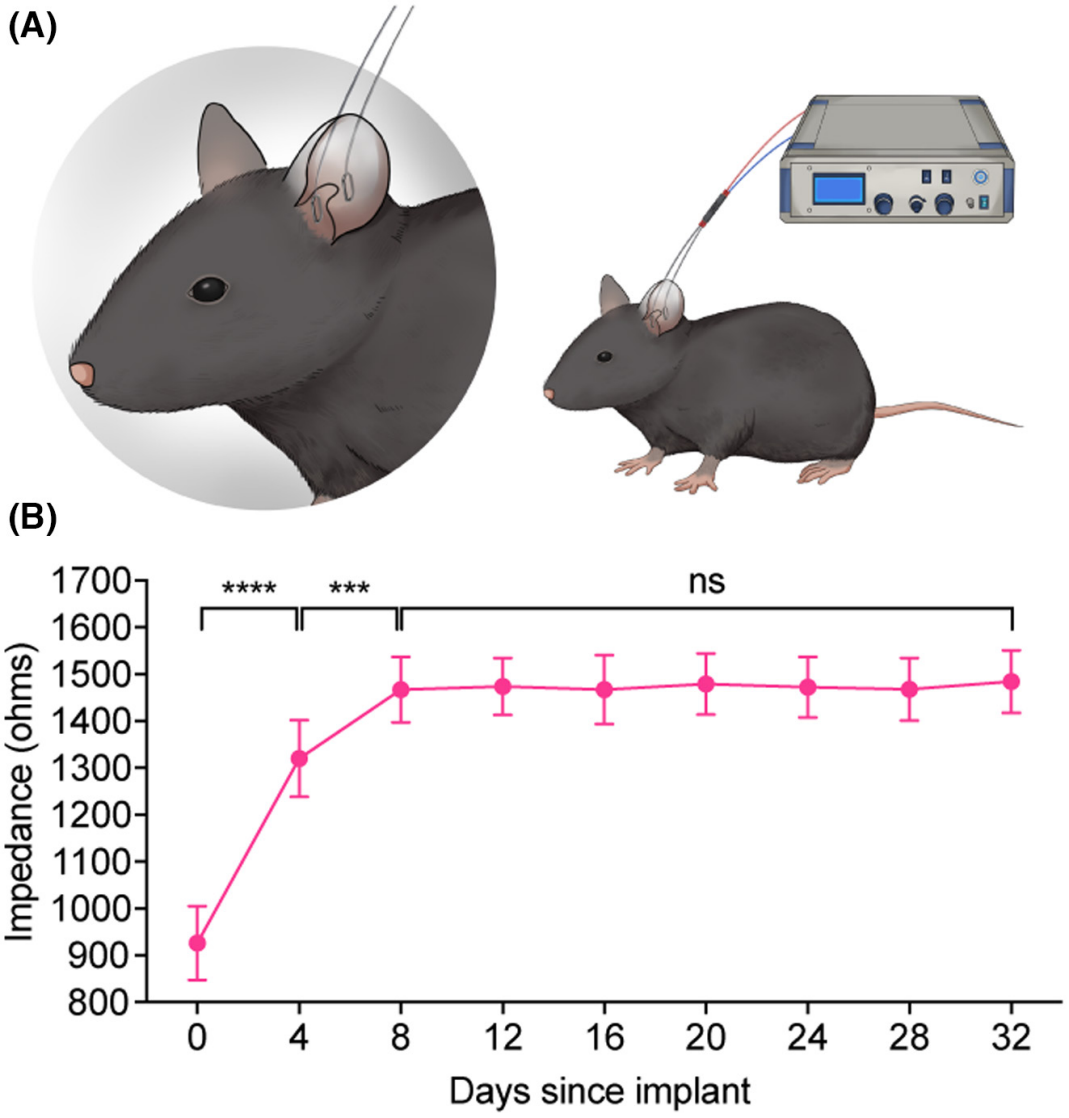
The diagram of electrode implantation of the taVNS device in awake mice. (A) Schematic representation of the method used to implant the taVNS device in the mouse auricle, which is the distribution area of the ABVN. (B) The electrode impedance data showed that the impedance was stable 8 days after electrode implantation. ***p < 0.001, ****p < 0.0001; ns, no significant difference (*p* > 0.05). The data were analyzed by paired two‐tailed *t*‐tests. The data are presented as the mean ± SD. ABVN, auricular branch of the vagus nerve; taVNS, transcutaneous auricular vagus nerve stimulation.

Mice were anesthetized with 1% sodium pentobarbital (40 mg/kg, intraperitoneal injection, i.p.) and placed on a heating pad to maintain their body temperature at 35–37°C, and then, their eyes were coated with ophthalmic ointment to prevent drying. To minimize impedance, the fur was removed from the electrode implantation site with a mild depilatory cream. Immediately, two 0.3‐mm‐diameter platinum wire electrodes were placed in the cymba conchae, which is innervated by the auricular branch of the vagus nerve (ABVN). The skin of the ear was penetrated with the platinum wires to form a loop (total length of approximately 1 cm, approximately 0.5 cm on the inner and outer sides of the ear, respectively) around the ear skin, and the platinum wire on the dorsal side of the ear and connected to a stimulator (Figure 1A).

### 2.3 Transcutaneous auricular vagus nerve stimulation (taVNS)

The electrodes were connected to a stimulator (Kedoubc, Suzhou, China) by wires (Figure 1A). The stimulation parameters were as follows: 0.8 mA, 20 Hz, the total length of 30 min, and a 300 μs pulse width.

### 2.4 Stereotaxic surgery and virus injection

**FIGURE 2:**
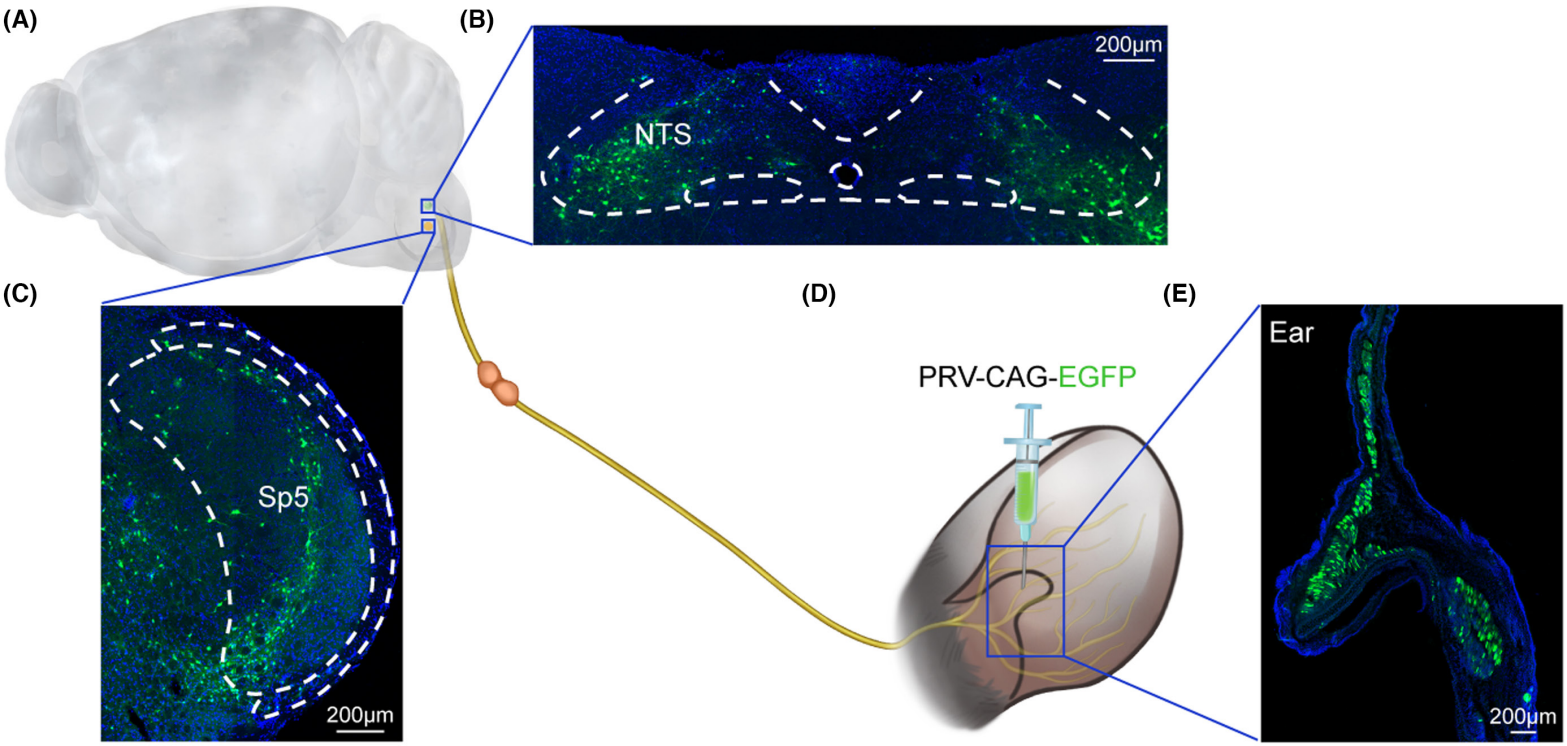
Schematic of validation of the reliability of the taVNS method by utilizing the retrograde neurotropic PRV tracing strategy. (A) Schematic representation of the location of brainstem nuclei NTS and Sp5. (B) Representative confocal image showing the EGFP‐positive neurons in the NTS. (C) Representative confocal image showing the EGFP‐positive neurons in the Sp5. (D) Schematic representation of injection of the retrograde PRV‐CAG‐EGFP virus into the helix of the mouse ear. (E) Representative confocal image showing the virus injection site in the ear. Scale bar: 200 μm. NTS, nucleus tractus solitarius; PRV, pseudorabies virus; Sp5, spinal trigeminal nucleus.

To verify the distribution of the vagus nerve at the electrode site and its connection with the brain nucleus, we anesthetized the mice with 1% pentobarbital (40 mg/kg, i.p.) and then fixed them on a stereotaxic apparatus (RWD, Shenzhen, China). Then, their eyes were lubricated with ophthalmic ointment. A total of 300–500 nL volume of PRV‐CAG‐EGFP (BrainVTA, Wuhan, China) was injected into the helix of the ear at a rate of 0.1 μL/min (Figure 2D). After injection, the needle was slowly retracted to minimize backflow. The mice were subsequently placed in a cage on a heating pad and returned to their home cages when they were fully awake. After allowing 5 days for virus expression, histological verification of the viral injection site and infection of target brain regions was performed (Figure 2A–C,E).

### 2.5 Immunohistochemistry

Mice were sacrificed in a CO_2_ chamber and then subjected to cardiac perfusion with 20 mL of phosphate‐buffered saline (PBS) followed by 20 mL of 4% paraformaldehyde (PFA). The mouse brains were removed, post‐fixed in PFA for 4–6 h, and subsequently dehydrated with 30% sucrose at 4°C until the tissue sank to the bottom. Coronal brain sections (30 mm thick) were prepared with a freezing microtome (VT1000S, Leica Microsystems). Confocal images were acquired using a confocal microscope (LSM 880, Carl Zeiss). To obtain images of entire sections, image tiles were stitched together using Zeiss software. The images were further processed using ZEN software (version 2.3, blue edition; Zeiss, Germany).

### 2.6 Open field test (OFT)

Mice were placed in a white plastic open field (40 cm × 40 cm × 50 cm), which was divided into a 3 × 3 central area, four corner areas, and four side areas. The trajectories of the mice over 5 min were recorded by the SMART tracking system (Panlab, Spain) to assess anxiety‐related behaviors.

### 2.7 Elevated plus maze (EPM) test

The EPM test is used to assess anxiety levels.^33^, ^34^ The maze was elevated 50 cm above the ground and consisted of two open arms (30 cm × 5 cm), two closed arms (30 cm × 5 cm, 20 cm tall walls) with open roofs, and a central platform (5 cm × 5 cm). The mice were placed on the central platform facing an open arm. They were allowed to freely explore the maze for 5 min, and their behavior was recorded and analyzed with EthoVision XT 14.0 software.

### 2.8 Novel object recognition test (NORT)

Mice were placed in a square arena (25 cm × 25 cm × 30 cm) with two identical objects in adjacent corners and allowed to freely explore the arena for 10 min. After 2 h of rest in their home cages, the mice were returned to the arena after one object was randomly replaced by a new object. The time spent exploring each object was recorded over 5 min to assess the memory of the mice.

### 2.9 Y‐maze test

The Y‐maze test was performed with a Y‐shaped device consisting of 3 white, closed arms at an angle of 120° to each other (A, B, and C), with each arm being 30 cm long, 6 cm wide, and 15 cm high. After closing arm C with a white plastic sheet, the mice were placed in arm A and allowed to freely explore arms A and B. After 2 h of rest in their home cages, the mice were returned to the arena. During the test, arm C was opened, and the exploratory behavior of the mice in the three arms over 5 minutes was analyzed.

### 2.10 Statistical analysis

The data are presented as the mean ± SD. All statistical analyses were performed with GraphPad Prism 7.0 (GraphPad Software, Inc., USA). Two‐way ANOVA and Bonferroni post hoc analyses were used in analyses with multiple comparisons. Paired two‐tailed *t*‐tests or unpaired two‐tailed *t*‐tests were used for comparison between two groups. *p* < 0.05 indicated statistical significance.

## 3 RESULTS

First, we evaluated the reliability of the placement of the taVNS device in the mouse ear. The impedance of the stimulating electrodes stabilized 8 days after implantation and was maintained throughout the 3‐week taVNS procedure (Figure 1B), suggesting that the attachment between the stimulating electrodes and the cymba conchae was stable. The auricular branch of the vagus nerve (ABVN) is a branch of the vagus nerve that forms a cutaneous receptive field over the auricle^35^, ^36^ and projects somatosensory afferents to the nucleus tractus solitarius (NTS) and spinal trigeminal nucleus (Sp5).^16^ By utilizing the retrograde neurotropic pseudorabies virus (PRV) tracing strategy,^37^, ^38^, ^39^, ^40^ we confirmed the connection of the ABVN with the NTS and Sp5, providing an anatomical basis for the reliable application of taVNS. PRV‐CAG‐EGFP was unilaterally injected into the helix of the mouse ear (Figure 2D). Five days later, we sacrificed the mice and observed EGFP‐labeled neurons in the NTS and Sp5 (Figure 2B,C).

**FIGURE 3:**
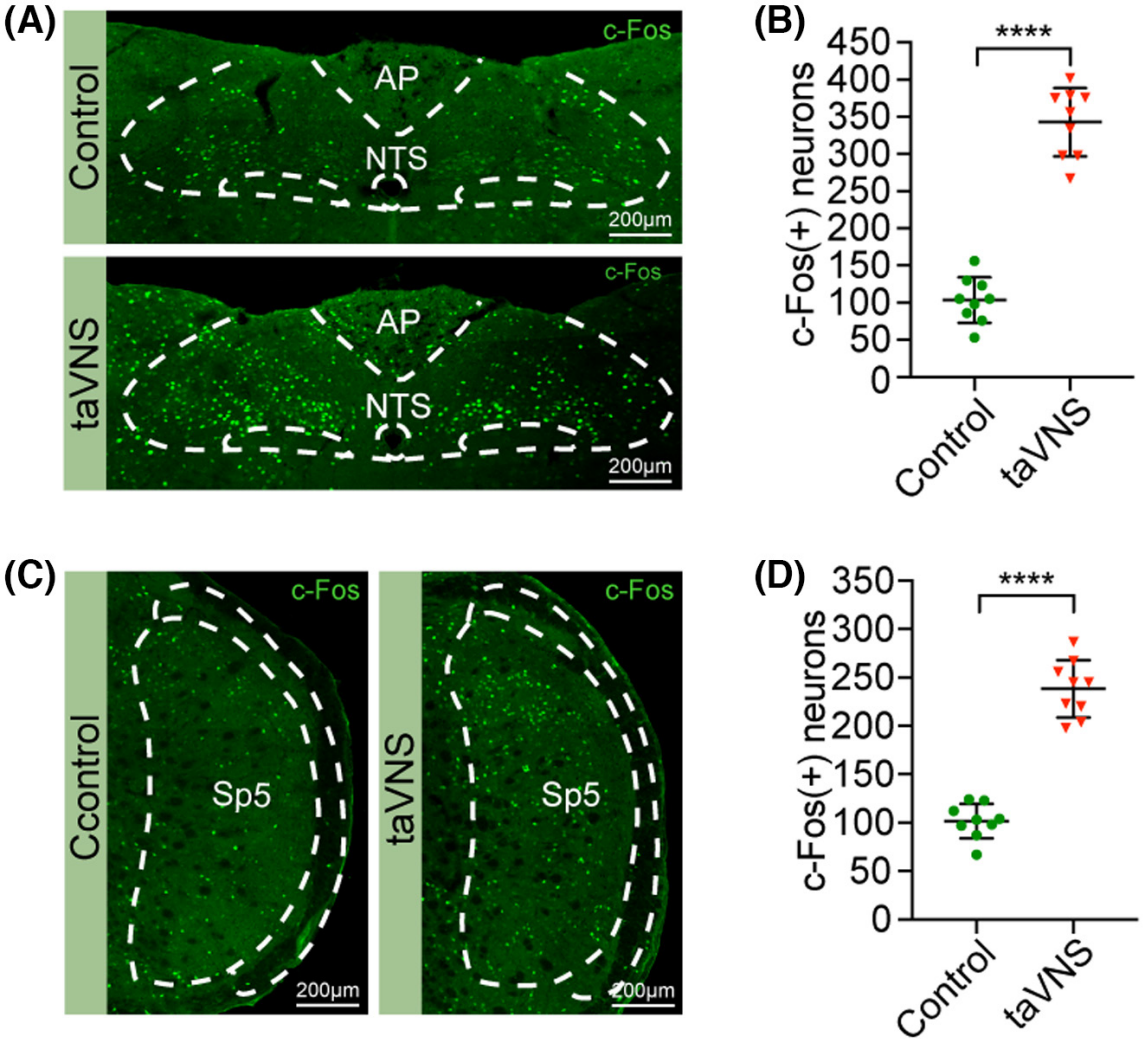
taVNS effectively activates the NTS and Sp5 in freely moving awake mice. (A) Representative images of c‐Fos‐positive neurons in the NTS in control and taVNS group mice. *n* = 9 sections from three mice per group. Scale bar: 200 μm. (B) The expression levels of c‐Fos in the NTS were significantly higher than those in the control group (control = 103.56 ± 30.59, taVNS = 342.78 ± 46.05, ****p < 0.0001). (C) Representative images of c‐Fos‐positive neurons in the Sp5 in control and taVNS group mice. *n* = 9 sections from three mice per group. Scale bar: 200 μm. (D) The expression levels of c‐Fos in the Sp5 were significantly higher than those in the control group (control = 101.67 ± 17.75, taVNS = 238.33 ± 29.51, ****p < 0.0001). The data were analyzed by unpaired two‐tailed *t*‐test. The data are presented as the mean ± SD. AP, area postrema.

**FIGURE 4:**
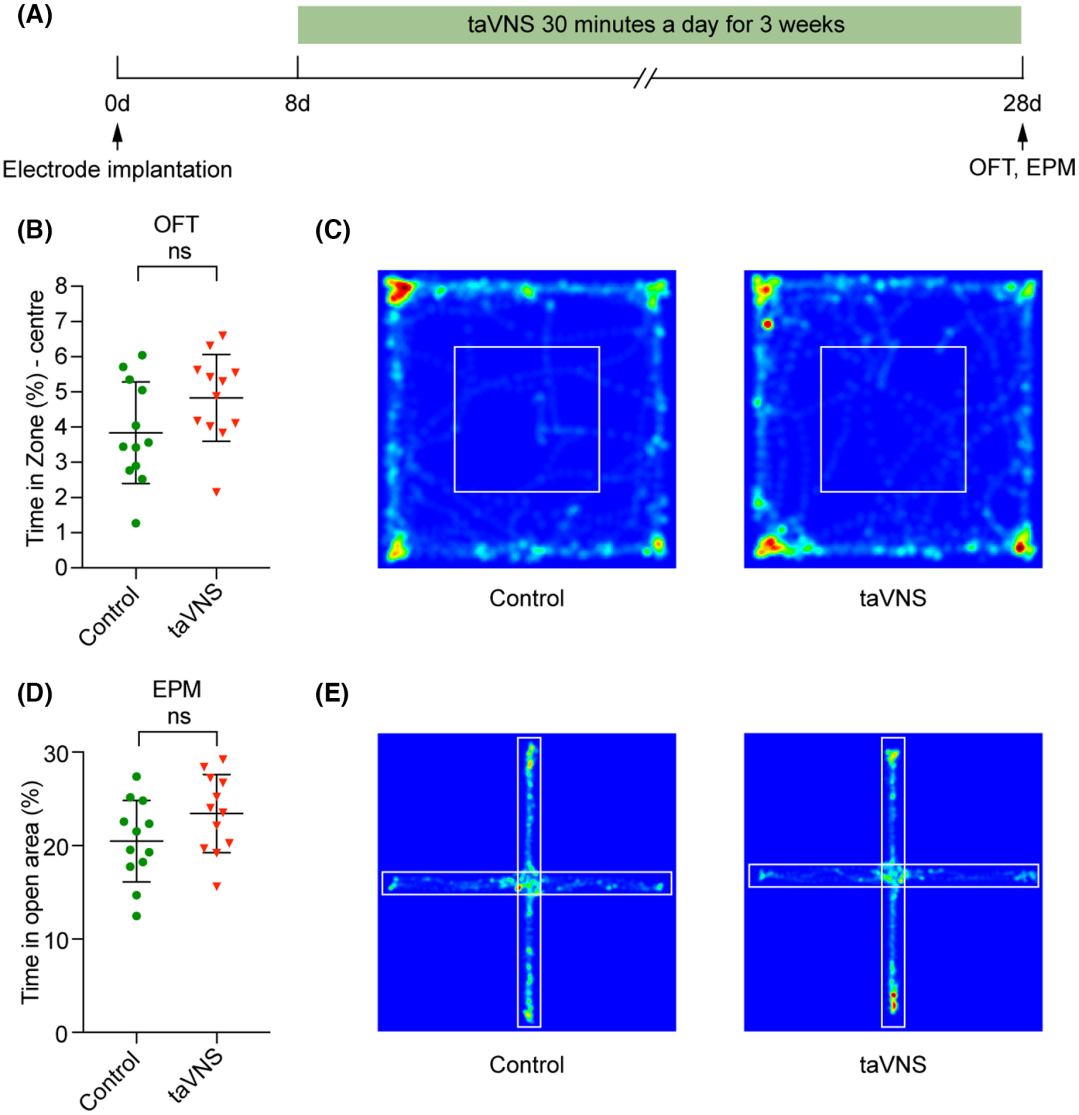
Application of taVNS for 3 weeks has no effect on anxiety‐like behaviors in freely moving mice. (A) Experimental scheme showing the application of taVNS and behavioral tests. (B) There was no significant difference between control and taVNS mice in the percentage of time spent in the central area in the OFT (control = 3.84 ± 1.44, taVNS = 4.83 ± 1.24, *p* = 0.0842). *n* = 12 mice/group. (C) Representative heat maps showed the time spent in the open field chamber of control and taVNS mice. (D) There was no significant difference between control and taVNS mice in the percentage of time spent in the open area in the EPM test (control = 20.48 ± 4.37, taVNS = 23.43 ± 4.18, *p* = 0.1051). *n* = 12 mice/group. (E) Representative heat maps showed the time spent in open arms of control and taVNS mice. ns, no significant difference (*p* > 0.05). The data were analyzed by unpaired two‐tailed *t*‐test. The data are presented as the mean ± SD. EPM, elevated plus maze; OFT, open field test.

**FIGURE 5:**
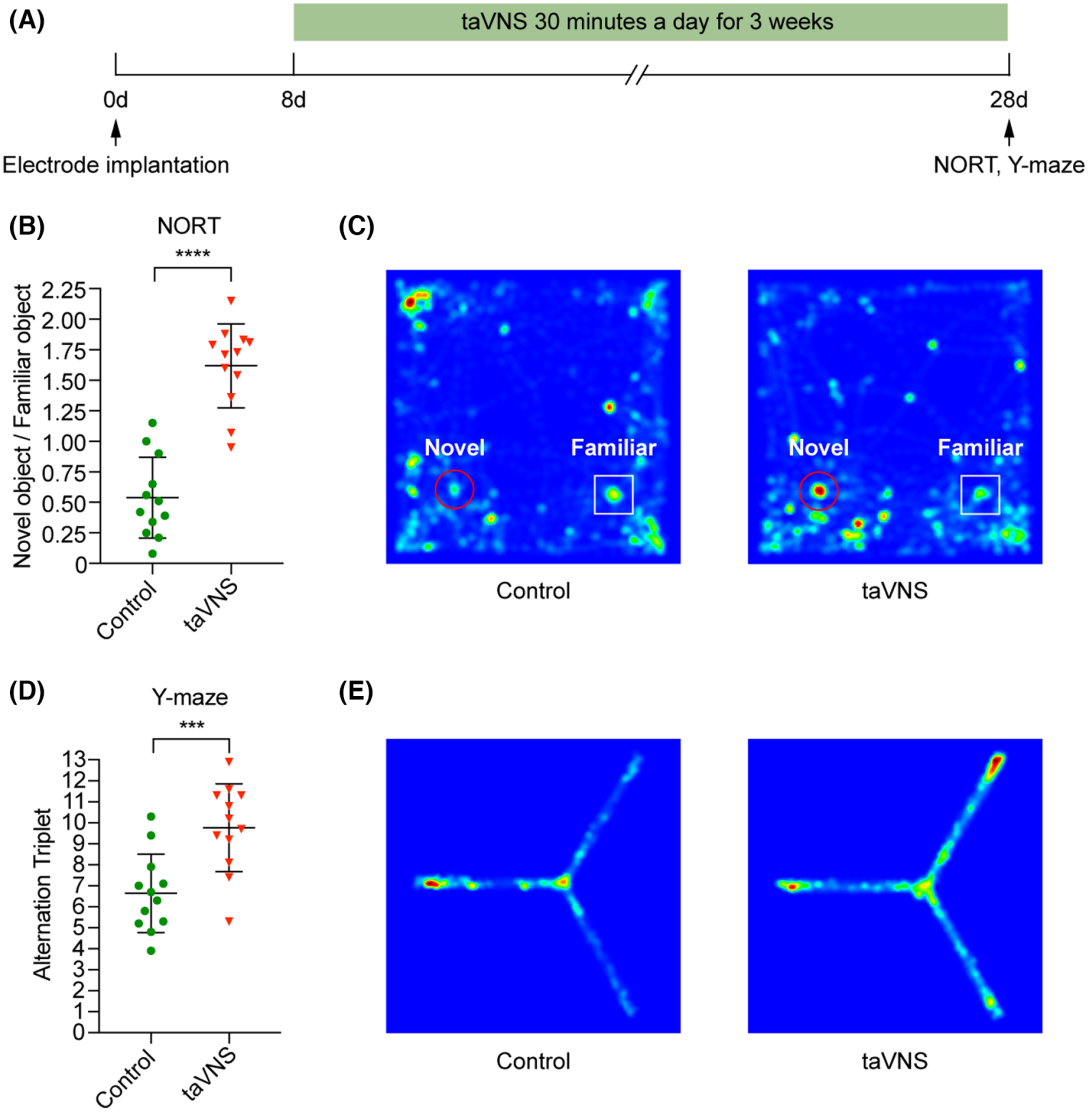
Application of taVNS for 3 weeks improves memory in freely moving mice. (A) Experimental scheme showing the application of taVNS and behavioral tests. (B) The exploration time ratio for the novel and familiar objects in the NORT was significantly higher in the taVNS group than in the control group (control = 0.54 ± 0.33, taVNS = 1.62 ± 0.34, ****p < 0.0001). *n* = 12 mice/group. (C) Representative heat maps showed the time spent exploring each object of control and taVNS mice. (D) There was a significant increase in the number of spontaneous alternations in the Y‐maze test in the taVNS group compared to the control group (control = 6.64 ± 1.87, taVNS = 9.77 ± 2.09, ***p = 0.0008). *n* = 12 mice/group. (E) Representative heat maps showed the exploratory behavior in the three arms of control and taVNS mice. The data were analyzed by unpaired two‐tailed *t*‐test. The data are presented as the mean ± SD. NORT, novel object recognition test.

We subsequently confirmed the effectiveness of this taVNS strategy. We assessed changes in the excitability of the NTS and Sp5 after taVNS by evaluating the expression of c‐Fos, a molecular proxy of neural activity.^41^ After 3 weeks of taVNS, the expression levels of c‐Fos in the NTS and Sp5 were dramatically higher than those in the control group (c‐Fos in the NTS: control = 103.56 ± 30.59, taVNS = 342.78 ± 46.05, *p* < 0.0001; c‐Fos in the Sp5: control = 101.67 ± 17.75, taVNS = 238.33 ± 29.51, *p* < 0.0001) (Figure 3). While taVNS was administered under anesthesia in previous studies,^2^, ^12^, ^28^, ^42^, ^43^ electrical stimulation was applied in awake mice in the present study; consequently, it was necessary to monitor anxiety‐like behaviors and body weight to exclude the possibility of negative emotions caused by an improper stimulus intensity. The time spent in the center or open area in the open field test (OFT) or elevated plus maze (EPM), respectively, did not significantly differ between the taVNS group and control group (OFT: control = 3.84 ± 1.44, taVNS = 4.83 ± 1.24, *p* = 0.0842; EPM: control = 20.48 ± 4.37, taVNS = 23.43 ± 4.18, *p* = 0.1051) (Figure 4). Compared with the control group mice, the taVNS group mice exhibited no significant differences in body weight (Figure S1). Consistent with previous findings,^25^ compared with the control group, the taVNS group showed a significant improvement in object recognition memory after 3 weeks of stimulation, which provides convincing evidence for the effect of taVNS on improving memory behaviors (novel object recognition test (NORT): control = 0.54 ± 0.33, taVNS = 1.62 ± 0.34, *p* < 0.0001; Y‐maze: control = 6.64 ± 1.87, taVNS = 9.77 ± 2.09, *p* = 0.0008) (Figure 5).

## 4 DISCUSSION

We report a new method for applying taVNS in awake mice that is safe, noninvasive, and well tolerated, and based on anatomical validation and improvements in memory, we confirm that this new stimulation modality is effective.

In a large number of previous clinical studies, taVNS was applied persistently and repetitively, often in awake patients, for safety and maneuverability reasons.^13^, ^44^ In contrast, in many animal experiments, mice are given general anesthesia when receiving taVNS due to technical and equipment limitations.^2^, ^3^, ^12^, ^25^, ^28^, ^42^, ^43^ Repeated anesthesia may have marked impacts on the brain, such as neurotoxicity and cognitive impairment.^7^ In the present study, taVNS was administered to awake mice, allowing basic research findings to be generalized to clinical situations and providing a technique for treating various diseases with taVNS in animals.

Platinum wires were used as the electrode for taVNS due to their excellent histocompatibility and corrosion resistance. The impedance of the stimulating electrodes remained stable approximately 1 week after implantation, which indicated that the device could be reliably fixed and had stable performance. Neuroanatomical studies have shown that the only branch of the vagus nerve on the surface of the body is the ABVN, and the cymba conchae is innervated exclusively by the ABVN.^12^, ^25^, ^45^ The NTS and Sp5, as the brainstem nuclei, in which vagus nerve afferent fibers terminate, are considered relay stations for visceral information, and they receive signals from the ear and regulate body functions.^3^, ^35^ The ABVN‐innervated region of the ear, known as the cymba conchae, was found to be clearly connected to the NTS and Sp5 in the brainstem through retrograde transsynaptic PRV tracing. This work further confirmed that the electrode implantation site was located in a dense region of the vagus nerve in the ear, providing an anatomical basis for the effects of taVNS.

Notably, the stimulus intensity used for taVNS was effective in freely moving awake mice, as evidenced by elevated c‐Fos expression in the NTS and Sp5. In the preliminary experiment, we monitored the behavior of mice receiving taVNS at different stimulus intensities and determined that 0.8 mA was the highest stimulus intensity that did not interfere with free movement in awake mice under the current experimental conditions. Consistent with the findings of previous studies, our present study showed that taVNS improved memory but did not induce anxiety in mice.^3^, ^25^ All mice tolerated taVNS without adverse events during the experiment (see Data S2).

Inevitably, there are certain limitations to this method. Because of the platinum wires connection between the mice's ears and the stimulator, some interference may occur if behavioral tests need to be performed at the same time as the taVNS. However, in general, this type of connection has little effect on mice because behavioral tests are rarely required at the time of stimulation. Of course, we tried to consider wireless stimulation to address this limitation. For example, wireless stimulation is performed by embedding an induction coil in the mouse, but in this case, the stability of the stimulation is difficult to ensure as the mouse position changes. A previous study applied wireless optoelectronic devices to vagus nerve stimulation in mice, but there were many drawbacks, such as invasiveness, limited efficiency, and large individual differences.^46^ Taken together, our current method is applicable to most studies.

## 5 CONCLUSION

This study developed a new taVNS method that utilizes a device that can be fully immobilized, fully mimics the taVNS technique used in clinical settings, and is well tolerated by freely moving awake mice. In addition, administration of taVNS to awake mice for 3 consecutive weeks significantly improved memory but had no effect on anxiety‐like behaviors. This new taVNS method has promising applications in research on awake animals and has the potential to improve the generalizability of basic research findings in clinical settings.

## AUTHOR CONTRIBUTIONS

**Yu‐Mei Yu:** Methodology, software, data curation, visualization, investigation, and writing—original draft. **Rui Yao:** Resources, data curation, investigation, and software. **Zhou‐Liang Liu:** Methodology, software, visualization, and investigation. **Yao Lu:** Methodology, resources, and investigation. **Yang‐Zi Zhu:** Conceptualization, supervision, investigation, formal analysis, resources, data curation, project administration, writing—original draft, and funding acquisition. **Jun‐Li Cao:** Conceptualization, supervision, writing—review and editing, writing—original draft, and funding acquisition.

## CONFLICT OF INTEREST STATEMENT

The authors declare no conflicts of interest.

## Supporting information


Data S1.

Data S2.

## Data Availability

The data sets generated and analyzed during the current study are available from the corresponding author upon reasonable request.

## References

[1] Redgrave J , Day D , Leung H , et al. Safety and tolerability of transcutaneous vagus nerve stimulation in humans: a systematic review. Brain Stimul. 2018;11(6):1225‐1238. 30217648 10.1016/j.brs.2018.08.010

[2] Li S , Wang Y , Gao G , et al. Transcutaneous auricular vagus nerve stimulation at 20 Hz improves depression‐like behaviors and Down‐regulates the hyperactivity of HPA Axis in chronic unpredictable mild stress model rats. Front Neurosci. 2020;14:680. 32765210 10.3389/fnins.2020.00680 PMC7378324

[3] Brambilla‐Pisoni C , Munoz‐Moreno E , Gallego‐Amaro I , et al. Auricular transcutaneous vagus nerve stimulation acutely modulates brain connectivity in mice. Front Cell Neurosci. 2022;16:856855. 35548372 10.3389/fncel.2022.856855 PMC9081882

[4] Wang L , Wang Y , Wang Y , et al. Transcutaneous auricular vagus nerve stimulators: a review of past, present, and future devices. Expert Rev Med Devices. 2022;19(1):43‐61. 34937487 10.1080/17434440.2022.2020095

[5] Ventureyra EC . Transcutaneous vagus nerve stimulation for partial onset seizure therapy. a new concept. Childs Nerv Syst. 2000;16(2):101‐102. 10663816 10.1007/s003810050021

[6] Pan L , Wang J , Wu W , Wang Y , Zhu Y , Song Y . Transcutaneous auricular vagus nerve stimulation improves working memory in temporal lobe epilepsy: a randomized double‐blind study. CNS Neurosci Ther. 2023;30(2):e14395. 37553557 10.1111/cns.14395 PMC10848055

[7] von Wrede R , Rings T , Schach S , Helmstaedter C , Lehnertz K . Transcutaneous auricular vagus nerve stimulation induces stabilizing modifications in large‐scale functional brain networks: towards understanding the effects of taVNS in subjects with epilepsy. Sci Rep. 2021;11(1):7906. 33846432 10.1038/s41598-021-87032-1 PMC8042037

[8] Mertens A , Gadeyne S , Lescrauwaet E , et al. The potential of invasive and non‐invasive vagus nerve stimulation to improve verbal memory performance in epilepsy patients. Sci Rep. 2022;12(1):1984. 35132096 10.1038/s41598-022-05842-3 PMC8821667

[9] Yang H , Shi W , Fan J , et al. Transcutaneous auricular vagus nerve stimulation (ta‐VNS) for treatment of drug‐resistant epilepsy: a randomized double‐blind clinical trial. Neurotherapeutics. 2023;20(3):870‐880. 36995682 10.1007/s13311-023-01353-9 PMC10275831

[10] Rong P , Liu A , Zhang J , et al. Transcutaneous vagus nerve stimulation for refractory epilepsy: a randomized controlled trial. Clin Sci (Lond). 2014; CS20130518. 10.1042/CS20130518 24684603

[11] Rong P , Liu A , Zhang J , et al. An alternative therapy for drug‐resistant epilepsy: transcutaneous auricular vagus nerve stimulation. Chin Med J. 2014;127(2):300‐304. 24438620

[12] Wang JY , Zhang Y , Chen Y , et al. Mechanisms underlying antidepressant effect of transcutaneous auricular vagus nerve stimulation on CUMS model rats based on hippocampal alpha7nAchR/NF‐kappaB signal pathway. J Neuroinflammation. 2021;18(1):291. 34920740 10.1186/s12974-021-02341-6 PMC8680337

[13] Fang J , Rong P , Hong Y , et al. Transcutaneous vagus nerve stimulation modulates default mode network in major depressive disorder. Biol Psychiatry. 2016;79(4):266‐273. 25963932 10.1016/j.biopsych.2015.03.025 PMC4838995

[14] Sun J , Guo C , Ma Y , et al. Immediate modulatory effects of transcutaneous auricular vagus nerve stimulation on the resting state of major depressive disorder. J Affect Disord. 2023;325:513‐521. 36642310 10.1016/j.jad.2023.01.035

[15] Chen Y , Zhang Y , Wang J , et al. Anti‐neuroinflammation effects of transcutaneous auricular vagus nerve stimulation against depression‐like behaviors via hypothalamic alpha7nAchR/JAK2/STAT3/NF‐kappaB pathway in rats exposed to chronic unpredictable mild stress. CNS Neurosci Ther. 2023;29(9):2634‐2644. 37032645 10.1111/cns.14207 PMC10401149

[16] Garcia RG , Lin RL , Lee J , et al. Modulation of brainstem activity and connectivity by respiratory‐gated auricular vagal afferent nerve stimulation in migraine patients. Pain. 2017;158(8):1461‐1472. 28541256 10.1097/j.pain.0000000000000930 PMC5517046

[17] Zhang Y , Huang Y , Li H , et al. Transcutaneous auricular vagus nerve stimulation (taVNS) for migraine: an fMRI study. Reg Anesth Pain Med. 2021;46(2):145‐150. 33262253 10.1136/rapm-2020-102088

[18] Straube A , Ellrich J , Eren O , Blum B , Ruscheweyh R . Treatment of chronic migraine with transcutaneous stimulation of the auricular branch of the vagal nerve (auricular t‐VNS): a randomized, monocentric clinical trial. J Headache Pain. 2015;16:543. 26156114 10.1186/s10194-015-0543-3 PMC4496420

[19] Chen SP , Ay I , Lopes de Morais A , et al. Vagus nerve stimulation inhibits cortical spreading depression. Pain. 2016;157(4):797‐805. 26645547 10.1097/j.pain.0000000000000437 PMC4943574

[20] Morais A , Liu TT , Qin T , et al. Vagus nerve stimulation inhibits cortical spreading depression exclusively through central mechanisms. Pain. 2020;161(7):1661‐1669. 32142015 10.1097/j.pain.0000000000001856 PMC7305968

[21] Wang L , Zhang J , Guo C , et al. The efficacy and safety of transcutaneous auricular vagus nerve stimulation in patients with mild cognitive impairment: a double blinded randomized clinical trial. Brain Stimul. 2022;15(6):1405‐1414. 36150665 10.1016/j.brs.2022.09.003

[22] De Smet S , Baeken C , Seminck N , et al. Non‐invasive vagal nerve stimulation enhances cognitive emotion regulation. Behav Res Ther. 2021;145:103933. 34332299 10.1016/j.brat.2021.103933

[23] Zhou Q , Zheng Z , Wang X , et al. taVNS alleviates sevoflurane‐induced cognitive dysfunction in aged rats via activating basal forebrain cholinergic neurons. Neurochem Res. 2023;48(6):1848‐1863. 36729311 10.1007/s11064-023-03871-6

[24] Ventura‐Bort C , Wirkner J , Wendt J , Hamm AO , Weymar M . Establishment of emotional memories is mediated by vagal nerve activation: evidence from noninvasive taVNS. J Neurosci. 2021;41(36):7636‐7648. 34281991 10.1523/JNEUROSCI.2329-20.2021 PMC8425981

[25] Vazquez‐Oliver A , Brambilla‐Pisoni C , Domingo‐Gainza M , Maldonado R , Ivorra A , Ozaita A . Auricular transcutaneous vagus nerve stimulation improves memory persistence in naive mice and in an intellectual disability mouse model. Brain Stimul. 2020;13(2):494‐498. 31919001 10.1016/j.brs.2019.12.024

[26] Sun JB , Cheng C , Tian QQ , et al. Transcutaneous auricular vagus nerve stimulation improves spatial working memory in healthy young adults. Front Neurosci. 2021;15:790793. 35002607 10.3389/fnins.2021.790793 PMC8733384

[27] Zhang H , Cao XY , Wang LN , et al. Transcutaneous auricular vagus nerve stimulation improves gait and cortical activity in Parkinson's disease: a pilot randomized study. CNS Neurosci Ther. 2023;29(12):3889‐3900. 37311693 10.1111/cns.14309 PMC10651956

[28] Lv H , Yu X , Wang P , et al. Locus coeruleus tyrosine hydroxylase positive neurons mediated the peripheral and central therapeutic effects of transcutaneous auricular vagus nerve stimulation (taVNS) in MRL/lpr mice. Brain Stimul. 2023;17(1):49‐64. 38145753 10.1016/j.brs.2023.12.008

[29] Wu J , Yang JJ , Cao Y , et al. Iron overload contributes to general anaesthesia‐induced neurotoxicity and cognitive deficits. J Neuroinflammation. 2020;17(1):110. 32276637 10.1186/s12974-020-01777-6 PMC7149901

[30] McCann ME , Soriano SG . Does general anesthesia affect neurodevelopment in infants and children? BMJ. 2019;367:l6459. 31818811 10.1136/bmj.l6459

[31] Vutskits L , Xie Z . Lasting impact of general anaesthesia on the brain: mechanisms and relevance. Nat Rev Neurosci. 2016;17(11):705‐717. 27752068 10.1038/nrn.2016.128

[32] Roque PS , Thorn Perez C , Hooshmandi M , et al. Parvalbumin interneuron loss mediates repeated anesthesia‐induced memory deficits in mice. J Clin Invest. 2023;133(2):e159344. 36394958 10.1172/JCI159344 PMC9843048

[33] File SE , Lippa AS , Beer B , Lippa MT . Animal tests of anxiety. Curr Protoc Neurosci. 2004;27: 5.38.1‐5.38.21. 10.1002/0471142301.ns0803s26 18428606

[34] Ji YW , Shen ZL , Zhang X , et al. Plasticity in ventral pallidal cholinergic neuron‐derived circuits contributes to comorbid chronic pain‐like and depression‐like behaviour in male mice. Nat Commun. 2023;14(1):2182. 37069246 10.1038/s41467-023-37968-x PMC10110548

[35] Wang Y , Li SY , Wang D , et al. Transcutaneous auricular vagus nerve stimulation: from concept to application. Neurosci Bull. 2021;37(6):853‐862. 33355897 10.1007/s12264-020-00619-y PMC8192665

[36] Kaniusas E , Kampusch S , Tittgemeyer M , et al. Current directions in the auricular vagus nerve stimulation II‐an engineering perspective. Front Neurosci. 2019;13:772. 31396044 10.3389/fnins.2019.00772 PMC6667675

[37] Fan L , Xiang B , Xiong J , He Z , Xiang H . Use of viruses for interrogating viscera‐specific projections in central nervous system. J Neurosci Methods. 2020;341:108757. 32371062 10.1016/j.jneumeth.2020.108757

[38] Feng MH , He ZG , Liu BW , et al. Parafascicular nucleus circuits: implications for the alteration of gastrointestinal functions during epileptogenesis. Epilepsy Behav. 2016;64:295‐298. 27773642 10.1016/j.yebeh.2016.07.022

[39] Ryu V , Watts AG , Xue B , Bartness TJ . Bidirectional crosstalk between the sensory and sympathetic motor systems innervating brown and white adipose tissue in male Siberian hamsters. Am J Physiol Regul Integr Comp Physiol. 2017;312(3):R324‐R337. 28077392 10.1152/ajpregu.00456.2015 PMC5401994

[40] Li J , Liu T , Dong Y , Kondoh K , Lu Z . Trans‐synaptic neural circuit‐tracing with neurotropic viruses. Neurosci Bull. 2019;35(5):909‐920. 31004271 10.1007/s12264-019-00374-9 PMC6754522

[41] Hunt SP , Pini A , Evan G . Induction of c‐fos‐like protein in spinal cord neurons following sensory stimulation. Nature. 1987;328(6131):632‐634. 3112583 10.1038/328632a0

[42] Choi S , Jang DC , Chung G , Kim SK . Transcutaneous auricular vagus nerve stimulation enhances cerebrospinal fluid circulation and restores cognitive function in the rodent model of vascular cognitive impairment. Cells. 2022;11(19):3019. 36230988 10.3390/cells11193019 PMC9564197

[43] Go YY , Ju WM , Lee CM , Chae SW , Song JJ . Different transcutaneous auricular vagus nerve stimulation parameters modulate the anti‐inflammatory effects on lipopolysaccharide‐induced acute inflammation in mice. Biomedicine. 2022;10(2):247. 10.3390/biomedicines10020247 PMC8869637 35203459

[44] Sclocco R , Garcia RG , Kettner NW , et al. The influence of respiration on brainstem and cardiovagal response to auricular vagus nerve stimulation: a multimodal ultrahigh‐field (7T) fMRI study. Brain Stimul. 2019;12(4):911‐921. 30803865 10.1016/j.brs.2019.02.003 PMC6592731

[45] Peuker ET , Filler TJ . The nerve supply of the human auricle. Clin Anat. 2002;15(1):35‐37. 11835542 10.1002/ca.1089

[46] Donahue MJ , Ejneby MS , Jakesova M , et al. Wireless optoelectronic devices for vagus nerve stimulation in mice. J Neural Eng. 2022;19(6):066031. 10.1088/1741-2552/aca1e3 36356313

