# Supplementary material for: Feasibility and effectiveness of transcutaneous auricular vagus nerve stimulation (taVNS) in awake mice
Source: CNS Neurosci Ther. 2024 Sep 11;30(9):e70043. doi: 10.1111/cns.70043 (PMC11388527; doi:10.1111/cns.70043)
Supplement: Supplementary file 1 — Data S1. [file CNS-30-e70043-s002.docx]

**Supplementary Figure**


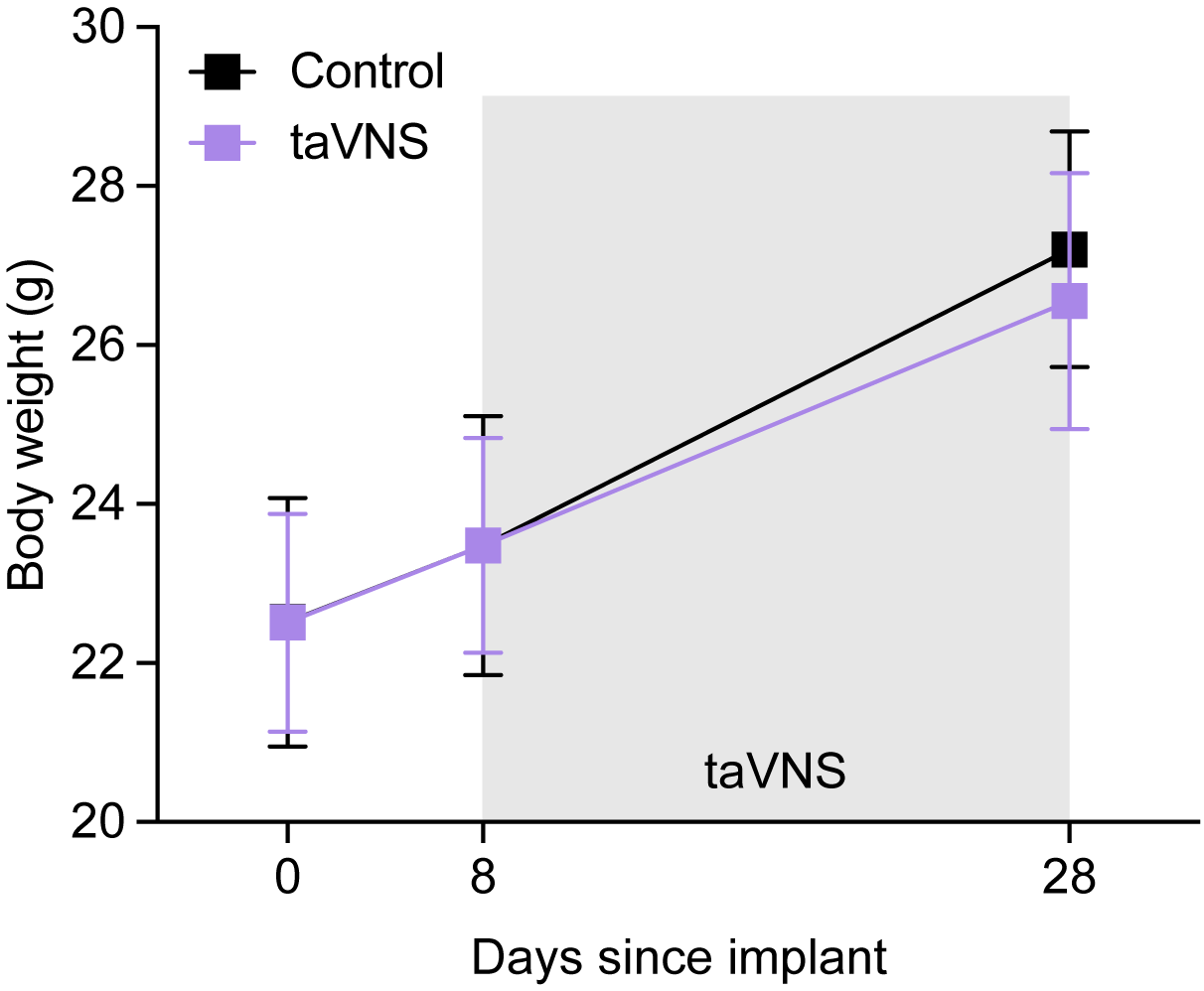


FIGURE S1. taVNS has no effect on body weight in normal mice.

Compared with the control group mice (black, n = 10 mice), the taVNS group mice (purple, n = 10 mice) exhibited no significant differences in body weight (Two-way ANOVA, *F*_1,18_ = 0.1151, *p* = 0.7384, post hoc Bonferroni test, data represented as mean ± SD).
